# Magnetic resonance-guided focused ultrasound subthalamotomy for Parkinson’s disease: a meta-analysis of effectiveness and safety

**DOI:** 10.1007/s10143-025-04045-4

**Published:** 2026-02-07

**Authors:** Abdallah Abbas, Haneen Sabet, Moaz Elsayed Abouelmagd, Mahmoud Tarek Hefnawy, Salma Bakr, Basant Lashin, Mohamed Ahmed Zanaty, Mohamed El-Moslemani, Mohamed Mohesn Helal, Majed Aldehri, Ibrahim Alnaami, Ahmed M. Raslan

**Affiliations:** 1 Faculty of Medicine, Al-Azhar University, Damietta, Egypt; 2 Faculty of Medicine, South Valley University, Qena, Egypt; 3 Faculty of Medicine, Cairo University, Cairo, Egypt; 4 Faculty of Medicine, Zagazig University, Zagazig, Egypt; 5 Department of Neurosurgery, Faculty of Medicine, Ain Shams University, Ain Shams, Egypt; 6 Department of Neurosurgery, Mayo Clinic Hospital, Rochester, MN USA; 7 Department of Anatomy, College of Medicine, King Khalid University, Abha, 8082, 62523 Saudi Arabia; 8 Department of Neurosurgery, Mental Health and Neuroscience, Maastricht University Medical Center, Maastricht, Netherlands; 9 Division of Neurosurgery, Department of Surgery, King Khalid University, Abha, Saudi Arabia; 10 Department of Neurological Surgery, Oregon Health & Science University, Portland, OR USA

**Keywords:** Focused ultrasound, Subthalamic nucleus, Parkinson's disease, Motor symptom treatment, Non-invasive neurosurgery

## Abstract

This systematic review and meta-analysis aimed to evaluate the safety and effectiveness of magnetic resonance-guided focused ultrasound (FUS) subthalamotomy in patients with Parkinson’s disease (PD), focusing on improvements in motor symptoms, quality of life (QoL), medication burden, and adverse events (AEs). Following PRISMA and Cochrane guidelines, a comprehensive search was conducted across PubMed, Cochrane CENTRAL, Scopus, and Web of Science up to November 2025. Studies were included if they assessed the safety or efficacy of unilateral or bilateral FUS-STN in PD. Data extraction and risk of bias assessment were independently conducted by two reviewers. Meta-analyses at six months used a random effects model to pool mean differences (MD) with 95% confidence intervals (CI). Five studies (*n* = 75) were included. FUS-subthalamic nucleus (STN) significantly reduced Movement Disorder Society-Sponsored Unified PD Rating Scale, Part III (MDS-UPDRS-III) scores at six months (MD: -11.05; 95% CI: [-14.68, -7.42]; *P* < 0.00001), with greater effects off-medication (*P* = 0.0009). Treated-side scores improved (MD: -10.67; 95% CI: [-13.41, -7.92]), while untreated-side scores did not. Significant improvements were also seen in MDS-UPDRS-II (MD: -3.58; 95% CI: [-4.84, -2.32]; *P* < 0.00001), PD Questionnaire-39 (PDQ-39) (MD: -8.55; 95% CI: [-13.51, -3.58]; *P* = 0.0007), and levodopa equivalent daily dose (LEDD) (MD: -111.63 mg; 95% CI: [-162.52, -60.74]; *P* < 0.0001). Common AEs included dyskinesia (9.2%), gait disturbance (6.9%), and dysarthria (6.4%). FUS-STN significantly improves motor function, daily living activities, and QoL (PDQ-39) and reduces dopaminergic medication burden in PD patients with asymmetrical symptoms. AEs were generally mild and manageable, supporting its role as a safe, effective, and non-incisional alternative to conventional surgical options.

**Supplementary Information:**

The online version contains supplementary material available at 10.1007/s10143-025-04045-4.

## Introduction

As therapeutic options evolve, non-incisional lesioning techniques such as focused ultrasound (FUS) have gained attention as emerging alternatives for managing motor symptoms in neurodegenerative disorders [1]. Parkinson’s disease (PD) is a progressive neurodegenerative disease characterized by rigidity, tremors, and bradykinesia with or without postural instability, secondary to loss of dopaminergic neurons of the substantia nigra pars compacta (SNpc) [2]. Treatment is primarily pharmacological using dopaminergic drugs; however, when motor symptoms become refractory to treatment or adverse events (AEs) are intolerable, ablative neurosurgical procedures and deep brain stimulation (DBS) are considered [2–5].

Surgical targets for PD include the ventral intermediate nucleus (Vim), globus pallidus internus (GPi), and the subthalamic nucleus (STN) [6, 7]. The Vim is primarily indicated for tremor-dominant phenotypes, as it effectively controls tremor but offers limited benefit for bradykinesia or rigidity. In contrast, both the GPi and STN have demonstrated positive effects on all the cardinal motor symptoms of the disease. The GPi is often preferred for patients with severe levodopa-induced dyskinesia due to its direct antidyskinetic effect, while the STN is more commonly chosen for ablative and neuromodulatory interventions due to its broader impact on symptoms [6–8]. Among the available targets for these interventions in PD, the STN stands out because it can modulate all major motor symptoms—tremor, rigidity, and bradykinesia—making it an especially attractive option [9–11].

Magnetic resonance-guided high-intensity FUS (MRgFUS) is one of the ablative tools in the neurosurgical armamentarium, and it is growing in popularity owing to its lack of invasiveness and comparable safety and efficacy to its more invasive counterparts, radiofrequency (RF) ablation and DBS [1]. Applying MRgFUS to the STN combines the functional advantages of this target with the incision-less profile of FUS, offering a compelling therapeutic option for patients unsuitable for or unwilling to undergo conventional surgery or device implantation [12]. The introduction of advanced imaging modalities has provided the benefit of real-time feedback through magnetic resonance imaging as well as clinical feedback to localize the intended lesions better [4, 12–15]. Despite the reversibility of the DBS, the non-incisional nature of MRgFUS has avoided complications possibly encountered with RF and DBS, including infection, intracranial hemorrhage, hardware malfunction, battery replacement, and cost [4, 6, 12, 13, 16]. Eligible patients were offered MRgFUS if they were not suited for surgery due to clinical contraindications or an aversion to undergoing invasive cranial surgery or hardware implantation [12–15, 17]. Despite that, the literature on unilateral and bilateral MRgFUS-STN for PD is still growing, but efficacy was believed to be comparable to other interventions on unilateral and bilateral STN lesions [18].

The goal of this systematic review and meta-analysis was to comprehensively evaluate the effectiveness and safety of MRgFUS-STN in patients with PD. Given the growing interest in less invasive and incisionless neurosurgical interventions and the limitations of current therapies, FUS-STN has emerged as a promising alternative for patients with asymmetrical motor symptoms. In addition to evaluating improvements in motor function, QoL, and medication burden, this study also assessed the incidence of AEs associated with the procedure, providing a balanced overview of its therapeutic benefits and potential risks. By synthesizing available clinical evidence, this study aims to clarify the role of FUS-STN and support informed clinical decision-making in PD management.

## Methods

We adhered to the PRISMA checklist and followed the guidelines outlined in the *Cochrane Handbook for Systematic Reviews of Interventions* [19, 20]*.*

### Search process and eligibility criteria

We performed a comprehensive search across four databases, Cochrane CENTRAL, PubMed, Web of Science, and Scopus, from their inception until November 2025, using the following search query: ((“Parkinson's disease” OR Parkinsonism OR PD OR Parkinson*) AND (“Focused ultrasound” OR FUS) AND (Subthalamotomy OR Subthalamic OR “Subthalamic nucleus” OR “Subthalamic lesion” OR STN)). For the detailed search strategy of each database, refer to Supplementary Table 1.

Our inclusion criteria encompassed all observational studies, clinical trials, and case series evaluating FUS-STN in patients with PD, specifically assessing safety, efficacy, or both. We excluded studies with different designs, including editorials, case reports, and abstracts. Additionally, studies investigating alternative targets, interventions, or outcomes were not considered.

### Study selection and data extraction

The study selection process was initially conducted at the title and abstract level, followed by a full-text review, using Rayyan software [21]. Each stage was performed by two independent authors, with any disagreements resolved by a third author.

Data collection was carried out using Microsoft Excel [22], with each study assessed by two independent authors. A third author acted as a comparator and resolved any discrepancies. The data collection sheet included the following information:

(i) **Summary data**: study ID, country, duration, study design, population, intervention, outcomes measured, and summary of the study.
(ii) **Baseline data**: sample size, age, sex, disease duration, baseline levodopa equivalent dose (LEDD; Levodopa Equivalent Daily Dose), most affected side, baseline Movement Disorder Society-Sponsored Unified PD Rating Scale, Part III (MDS-UPDRS-III) total score, and baseline PD Questionnaire-39 (PDQ-39).
(iii) **Efficacy outcomes**: MDS-UPDRS-III, MDS-UPDRS-II, PDQ-39, and LEDD.
(iv) **Safety outcomes**: mild AEs, moderate AEs, severe AEs, dyskinesia, gait disturbance, dysarthria, paresthesia, weakness, facial asymmetry, weight gain, nausea, dizziness, behavioral changes, and headache.

### Outcome measures

All outcomes were prespecified. The primary outcome for this meta-analysis was the change in MDS-UPDRS-III total score at six months post-procedure and the change in treated-side and untreated-side MDS-UPDRS-III motor scores. Secondary outcomes included change in MDS-UPDRS Part II (Activities of Daily Living) scores, change in PDQ-39 scores, reflecting QoL, change in the LEDD, and incidence and type of AEs.

### Bias risk assessment

For single-arm clinical trials, we used the MINORS tool [23], while for randomized controlled trials (RCTs), we applied the RoB 2.0 tool [24]. Additionally, for case series, we utilized the Joanna Briggs Institute (JBI) tool [25]. Details on the scoring system and algorithm of these tools can be found in the references.

### Statistical analysis

We conducted our analysis using RevMan version 5.4 and OpenMeta-Analyst [26]. To evaluate changes from baseline, we pooled the mean difference (MD) along with its 95% confidence interval (CI) using the generic inverse variance method, employing the DerSimonian and Laird random effects model [27]. This approach assumes that the included studies represent a random sample from a broader population, assigning greater weight to smaller studies. We opted for this model as it accounts for variability in the pooled estimate, making it particularly appropriate for datasets with potential inconsistencies or variations.

For baseline change calculations, we utilized the Meta-Analysis Accelerator tool [28]. To ensure accuracy, we estimated the correlation coefficient (CC) based on data from Martínez-Fernández [14], which provided both pre- and post-intervention values along with baseline changes. The estimated CC values were as follows: 0.291 for MDS-UPDRS-III total, 0.150 for MDS-UPDRS-III treated side, 0.716 for MDS-UPDRS-III untreated side, 0.735 for MDS-UPDRS-II, 0.581 for PDQ-39, and 0.93 for LEDD. The CCs were derived from Martínez-Fernández et al. [14], as this was the only included study that provided complete granular data (baseline mean/standard deviation (SD), final mean/SD, and change mean/SD) necessary for empirical calculation. This approach aligns with the *Cochrane Handbook for Systematic Reviews of Interventions* (Section 6.5.2.8), which prioritizes using correlations derived from similar studies over arbitrary constants [29]. This method was particularly critical for the MDS-UPDRS-III outcome, where we calculated distinct correlations for the treated side (r = 0.150) versus the untreated side (r = 0.716). These values reflect the clinical reality that surgical intervention disrupts the correlation between baseline and post-treatment scores on the treated side, whereas the untreated side follows the natural, more predictable progression of the disease.

To enhance accuracy and minimize heterogeneity, we selected the six-month endpoint for analysis. However, one study (Martínez-Fernández) [17] had a four-month endpoint, whereas the other four studies used a six-month endpoint.

We defined data heterogeneity based on a chi-square test p-value of less than 0.1 and an I^2^ statistic exceeding 50% [30]. When significant heterogeneity was detected, we conducted a leave-one-out analysis, sequentially excluding individual studies to identify the primary source of variation [31]. To further ensure the robustness of our findings and address potential sources of clinical method heterogeneity, we conducted two targeted sensitivity analyses. First, we re-analyzed the pooled data after excluding the study involving bilateral FUS-STN (Martínez-Fernández et al., 2020). Second, we performed a sensitivity analysis excluding the study with a four-month endpoint (Martínez-Fernández et al., 2020) to confirm that pooling it with six-month data did not introduce significant time-dependent bias into the primary efficacy results.

## Results

### Search and screening

**Fig. 1:**
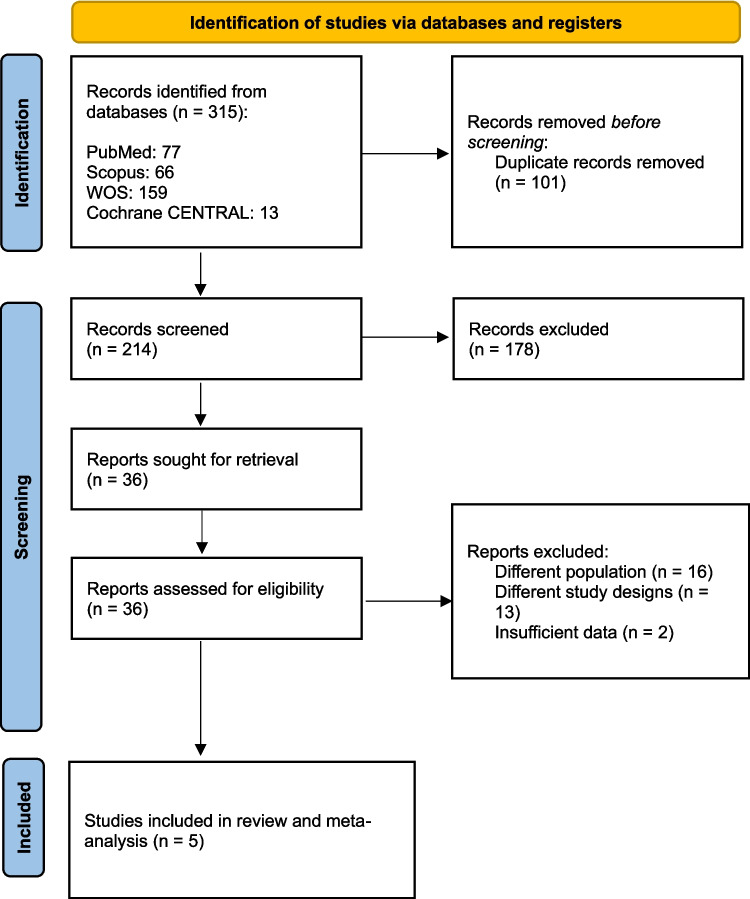
PRISMA flow diagram of study selection. Of 315 studies identified, 214 remained after duplicates were removed. Following title/abstract screening and full-text review, five studies met the inclusion criteria and were included in the meta-analysis evaluating MR-guided focused ultrasound subthalamotomy (FUS-STN) for Parkinson’s disease

A total of 315 studies were initially retrieved from four databases. After removing duplicates, 214 unique records remained. Title and abstract screening further reduced this number to 36, and following a full-text assessment, five studies [12–15, 17] met the inclusion criteria and were included in this systematic review and meta-analysis (see Fig. 1).

### Summary and baseline characteristics

The five studies included four conducted in Spain and one in the United Kingdom. The study designs consisted of three prospective nonrandomized studies of intervention (NRSI), one case series, and one RCT. The intervention used was MRgFUS-STN, which was unilateral in four studies and bilateral in one. For more details on the population inclusion criteria, measured outcomes, and study summaries, refer to Table 1.

**Table 1:** Summary of the included studies

Study ID	Country	Duration	Study design	Population	Intervention	Outcomes measured	Summary of the study
Campins-Romeu 2024	UK	April 2019 to October 2023	Prospective, single-center, open-label study	Asymmetric PD patients aged > 18 years old with poorly controlled motor signs on the affected side despite optimal dopaminergic therapy	**Unilateral** MR guided high-intensity FUS-STN	UPDRS III score change in off medication, Frequency and severity of AEs, Change in MDS-UPDRS III (treated side, On-medication), MDS-UPDRS III sub-scores (rigidity, bradykinesia, tremor, gait, axial; more affected side, Off/On-medication), general motor condition (MDS-UPDRS III total, Off/On-medication), activities of daily living (MDS-UPDRS II), motor complications (MDS-UPDRS IV), quality of life (EQ-5D, PDQ-39)	This study showed improvement in MDS-UPDRS III score, DS-UPDRS II, MDS-UPDRS IV, EQ-5D and PDQ-39, rigidity, bradykinesia, tremor and rest and decrease in LEDD doses and mild AEs
Armengou‐Garcia 2024	Spain	January 2020 to November 2021	Prospective, single-center, open-label study	Asymmetric PD patients aged > 30 years with at least 33% Improvement in UPDRS-III after levodopa challenge	**Unilateral** MR guided FUS-STN	UPDRS III score change in off medication, Frequency and severity of AEs, Change in Rigidity, Total tremor, Postural tremor, kinetic tremor, change in the LEDD, change in cognitive and neuropsychiatric tests, and the PGI-C	This study showed improvement in MDS-UPDRS III score, rigidity, bradykinesia, total tremor, rest, postural tremor, and kinetic tremor on the treated side and decrease in LEDD doses and mild AEs
Martínez-Fernández 2024	Spain	June 2019 to November 2023	Prospective, open-label, case series	Patients with PD who had received unilateral FUS-STN at least 6 months previously were recruited	**Bilateral** staged MR-guided FUS-STN	Safety (incidence and severity of AEs after the second treatment) and effectiveness in terms of motor change (measured with MDS-UPDRS III), motor complications (measured with the MDS-UPDRS IV), daily living activities (measured with the MDS-UPDRS I-II), quality of life (measured with PDQ-39), change in dopaminergic treatment, PCI-C (measured with PGI-C scale)	In this open-label case series, staged bilateral FUS-STN was associated with significant motor improvement among patients with PD. AEs were mostly mild and transient
Martínez‑Fernández 2020	Spain	March 2018 to May 2019	RCT	Patients with markedly asymmetric parkinsonism (asymmetry index > 1.5)	**Unilateral** MR-guided FUS-STN	Change from baseline to 4 months in MDS–UPDRS III, procedure-related complications (Safety), change in MDS–UPDRS IV, Unified dyskinesia rating scale, MDS–UPDRS I and, MDS–UPDRS II. Quality of life measured by PDQ-39, LEDD	FUS-STN in one hemisphere improved motor features of PD in patients with asymmetric signs with some AEs such as speech and gait disturbances, weakness on the treated side, and dyskinesia
Martínez-Fernández 2018	Spain	April 26, 2016 to June 14, 2016	Prospective, open-label single-arm pilot study	Patients with markedly asymmetric parkinsonism (asymmetry index ≥ 2)	**Unilateral** MR-guided FUS-STN	Safety outcomes at 1, 3, and 6 months after treatment, change in (MDS–UPDRS III) in both off-medication and on-medication states at 6 months, change in MDS–UPDRS IV, Unified dyskinesia rating scale, MDS–UPDRS I and MDS–UPDRS II. Quality of life measured by PDQ-39, LEDD	Unilateral MRI-guided FUS-STN was well tolerated and seemed to improve motor features of PD in patients with noticeably asymmetric parkinsonism

*PD*, Parkinson's disease; *MDS-UPDRS*, Movement disorder society unified parkinson's disease rating scale; *UPDRS*, Unified parkinson's disease rating scale; *MR*, Magnetic resonance; FUS, sFocused Ultrasound; *STN*, Subthalamic Nucleus; AEs, Adverse Events; *LEDD*, Levodopa equivalent daily dose; *PGI-C*, Patient’s global impression of change; *EQ-5D*, EuroQol five-dimension scale; *PDQ-39*, Parkinson’s disease questionnaire-39

The combined sample size across the five studies was 75, with an average age of 58.94 years (SD = 9.15). The sample included 52 males and 23 females. The mean disease duration was 6.61 years (SD = 3.22). For further details on LEDD, the most affected side, baseline MDS-UPDRS-III, and baseline PDQ-39, please refer to Table 2.

**Table 2:** Baseline characteristic of the study population

Study ID	Sample size (n)	Age, Years, Mean (SD)	Sex, Male (n): female (n)	Disease duration, years, mean (SD)	Baseline levodopa equivalent dose (LEDD), mg, Mean (SD)	Most affected body side (hemibody), n (%)	Baseline MDS-UPDRS Ill total score		Baseline PDQ-39
							Off-medication state	On-medication state	
Campins-Romeu 2024	12	60.47 (10.48)	9: 3	7.67 (2.52)	784 (213)	Right: 8 (80%)*	43.80 (13.84)	21 (14.26)	25.30 (18.03)
Armengou‐Garcia 2024	20	61.77 (7.34)	18: 2	6.83 (4.31)	615.83 (289.22)	Right: 12 (60%)	33.07 (10.53)	NA	NA
Martínez-Fernández 2024	6	56.07 (8.96)	3: 3	8.87 (3.15)	606.23 (380.24)	NA	NA	NA	8.53 (9.44)
Martínez‑Fernández 2020	27	56.6 (9.3)	16: 11	5.6 (2.5)	729.7 (328.3)	Right: 16 (59.3%)*	39.9 (9.7)	26.9 (6.7)	21.7 (12.0)
Martínez-Fernández 2018	10	59.5 (10.1)	6: 4	6.3 (2.5)	732.7 (346.4)	Left: 6 (60%)	32.7 (5.4)	21.5 (6.3)	12.6 (8.8)

*LEDD*, Levodopa equivalent daily dose; *MDS-UPDRS III*, Movement disorder society-sponsored unified parkinson’s disease rating scale, Part III; *PDQ-39*, Parkinson's disease questionnaire, a 39-item quality of life measure; *NA*, Not available; *Body-side laterality inferred assuming contralateral effects of STN subthalamotomy

### Risk of bias assessment

Three studies were assessed using the MINORS tool; two [13, 14] scored 14/16, and one [12] scored 13/16. Domain-level appraisal indicated that all three studies had limitations in blinding of outcome assessment, while additional concerns included shorter follow-up (Campins-Romeu 2024), incomplete description of attrition justification (Armengou-Garcia 2024), and lack of prospective sample-size calculation in two studies. The RCT [17], evaluated with RoB 2.0, showed low risk of bias across all domains, including randomization, deviations from intended intervention, missing data, outcome measurement, and selective reporting. The case series [15], assessed using the JBI tool, demonstrated low overall risk, with the only unclear domain being consecutive participant inclusion. Detailed scoring is available in Supplementary Tables 2–4.

### Analysis of primary outcomes

#### MDS-UPDRS-III at six months

**Fig. 2:**
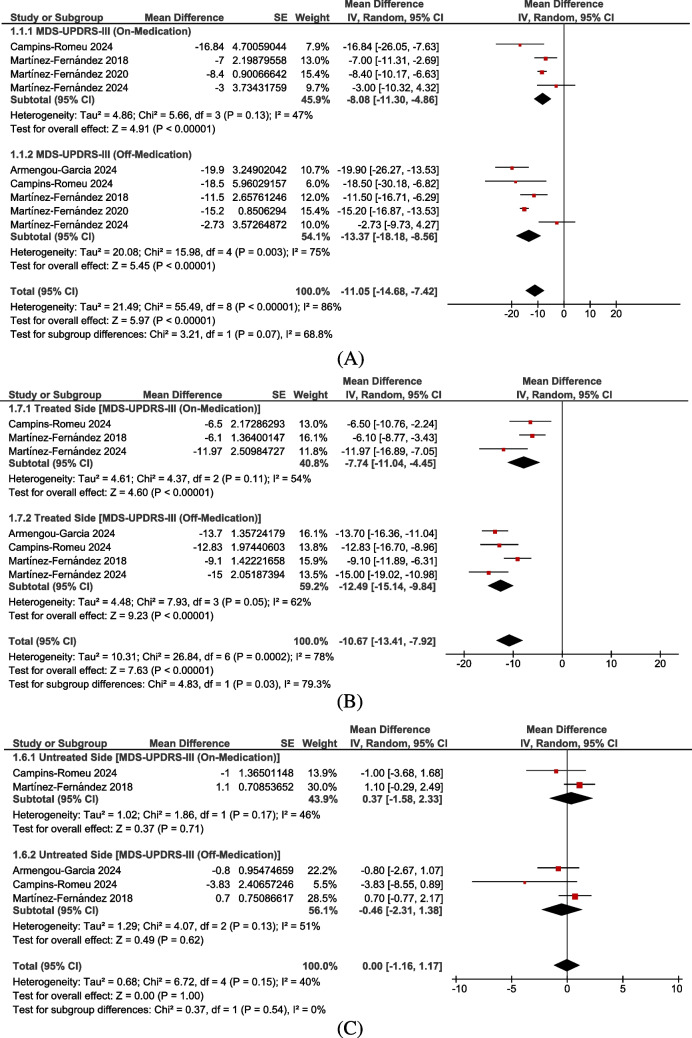
Forest plots of changes in MDS-UPDRS-III scores at six months. **A** Total MDS-UPDRS-III scores showed significant improvement (MD: –11.05; 95% CI: –14.68 to –7.42; *P* < 0.00001). **B** Treated-side scores improved significantly (MD: –10.67; 95% CI: –13.41 to –7.92; *P* < 0.00001), while. **C** Untreated-side scores showed no change (MD: 0.00; 95% CI: –1.16 to 1.17; *P* = 1.00)

The overall analysis revealed a significant reduction in MDS-UPDRS-III scores in patients who underwent FUS-STN compared to their pretreatment values (MD: −11.05 points, 95% CI: [−14.68, −7.42], *P* < 0.00001). However, there was significant overall heterogeneity (I^2^ = 86%, *P* < 0.00001) (Fig. 2A).

A subgroup analysis was performed for patients in the on-medication and off-medication states. Both subgroups showed significant reductions: (MD: −8.08 points, 95% CI: [−11.30, −4.86], *P* < 0.00001) for the on-medication group and (MD: −13.37 points, 95% CI: [−18.18, −8.56], *P* < 0.00001) for the off-medication group. Heterogeneity was moderate in the on-medication subgroup (I^2^ = 47%, *P* = 0.13) but high in the off-medication subgroup (I^2^ = 75%, *P* = 0.003) (Fig. 2A).

To address this heterogeneity, a leave-one-out analysis was conducted for the off-medication subgroup. Martínez-Fernández 2024 was identified as the primary source of heterogeneity. After excluding this study, heterogeneity was significantly reduced (I^2^ = 31%, *P* = 0.23), and the results remained significant (MD: −15.33 points, 95% CI: [−18.16, −12.51], *P* < 0.00001) (Supplementary Fig. 1).

After resolving heterogeneity, we compared the reduction in MDS-UPDRS-III scores between the on-medication and off-medication subgroups. The reduction was significantly greater in the off-medication group than in the on-medication group (*P* = 0.0009) (Supplementary Fig. 1).

Another sensitivity analysis was done after omitting the Martínez-Fernández et al., 2020 study, which had a four-month endpoint. The MDS-UPDRS-III was found to be −8.25 points in the on-medication group and −12.79 points in the off-medication group (refer to Supplementary Fig. 3).

Additional sensitivity analysis after omitting the Martínez-Fernández et al., 2024 study, which was bilateral. The MDS-UPDRS-III was found to be −8.84 points in the on-medication group and −15.33 points in the off-medication group, with a more significant reduction in the off-medication group (*P* = 0.003) (refer to Supplementary Fig. 4).

#### MDS-UPDRS-III in the treated side at six months

The overall analysis showed a significant reduction in MDS-UPDRS-III scores on the treated side in patients who underwent FUS-STN compared to their pretreatment values (MD: −10.67 points, 95% CI: [−13.41, −7.92], *P* < 0.00001). However, there was significant overall heterogeneity (I^2^ = 78%, *P* = 0.0002) (Fig. 2B).

A subgroup analysis was performed for patients in the on-medication and off-medication states. Both subgroups showed significant reductions: (MD: −7.74 points, 95% CI: [−11.04, −4.45], *P* < 0.00001) for the on-medication group and (MD: −12.49 points, 95% CI: [−15.14, −9.84], *P* < 0.00001) for the off-medication group. Heterogeneity was not significant in the on-medication subgroup (I^2^ = 54%, *P* = 0.11) but remained high in the off-medication subgroup (I^2^ = 62%, *P* = 0.05) (Fig. 2B).

To address this heterogeneity, a leave-one-out analysis was conducted for the off-medication subgroup. Martínez-Fernández 2018 was identified as the primary source of heterogeneity. After excluding this study, heterogeneity was significantly reduced (I^2^ = 0%, *P* = 0.75), and the results remained significant (MD: −13.78 points, 95% CI: [−15.71, −11.86], *P* < 0.00001) (Supplementary Fig. 2).

We compared the reduction in MDS-UPDRS-III scores between the on-medication and off-medication subgroups. The reduction was significantly greater in the off-medication group than in the on-medication group, both before (*P* = 0.03) and after addressing heterogeneity (*P* = 0.002) (Supplementary Fig. 2).

#### MDS-UPDRS-III in the untreated side at six months

The overall analysis did not find a significant reduction in MDS-UPDRS-III scores on the untreated side in patients who underwent FUS-STN compared to their pretreatment values (MD: 0.00 points, 95% CI: [−1.16, 1.17], *P* = 1.00). There was no significant heterogeneity (I^2^ = 40%, *P* = 0.15) (Fig. 2C).

A subgroup analysis was performed for patients in the on-medication and off-medication states. Both subgroups showed insignificant reductions: (MD: 0.37 points, 95% CI: [−1.58, 2.33], *P* = 0.71) for the on-medication group and (MD: −0.46 points, 95% CI: [−2.31, 1.38], *P* = 0.62) for the off-medication group.

### Analysis of secondary outcomes

#### MDS-UPDRS-II at six months

**Fig. 3:**
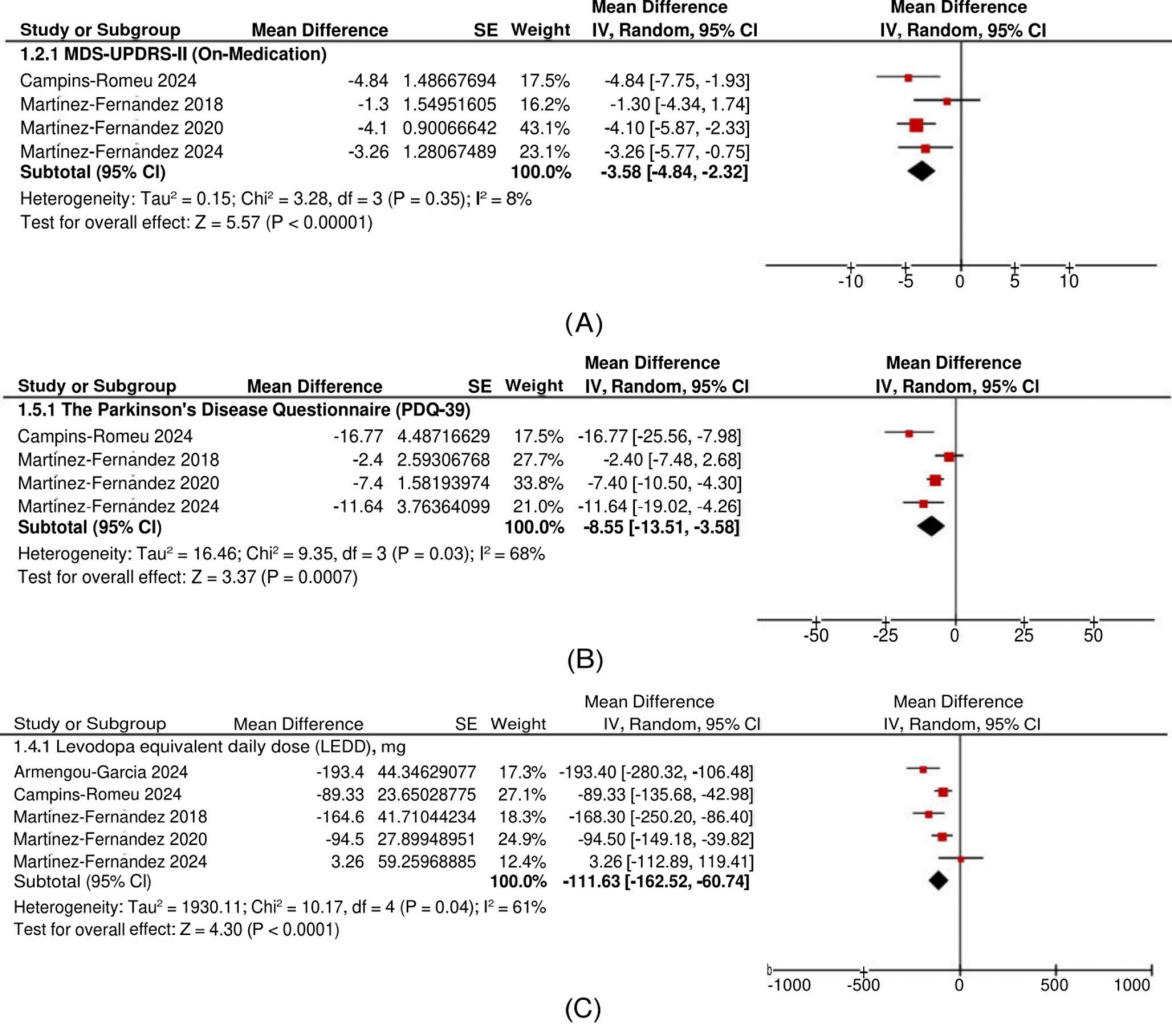
Forest plots of secondary outcomes at six months. **A** MDS-UPDRS-II scores decreased significantly (MD: –3.58; 95% CI: –4.84 to –2.32; *P* < 0.00001), indicating improved daily function. **B** PDQ-39 scores improved (MD: –8.55; 95% CI: –13.51 to –3.58; *P* = 0.0007), reflecting better quality of life. **C** Levodopa equivalent daily dose (LEDD) was reduced (MD: –111.63 mg; 95% CI: –162.52 to –60.74; *P* < 0.0001), suggesting decreased dopaminergic medication dependency following FUS-STN

The overall analysis revealed a significant reduction in MDS-UPDRS-II scores in patients in the on-medication state who underwent FUS-STN compared to their pretreatment values (MD: −3.58 points, 95% CI: [−4.84, −2.32], *P* < 0.00001). There was no significant overall heterogeneity (I^2^ = 8%, *P* = 0.35) (Fig. 3A).

#### PDQ-39 at six months

The overall analysis showed a significant reduction in PDQ-39 scores in patients who underwent FUS-STN compared to their pretreatment values (MD: −8.55 points, 95% CI: [−13.51, −3.58], *P* = 0.0007). However, there was significant overall heterogeneity (I^2^ = 68%, *P* = 0.03) (Fig. 3B).

To address this heterogeneity, a leave-one-out analysis was performed. Martínez-Fernández 2018 was identified as the primary source of heterogeneity. After excluding this study, heterogeneity was significantly reduced (I^2^ = 56%, *P* = 0.11), and the results remained significant (MD: −10.72 points, 95% CI: [−16.07, −5.36], *P* < 0.0001) (Supplementary Fig. 5).

#### LEDD (mg) at six months

The overall analysis revealed a significant reduction in LEDD in patients who underwent FUS-STN compared to their pretreatment values (MD: −111.63 mg, 95% CI: [−162.52, −60.74], *P* < 0.0001). However, there was significant overall heterogeneity (I^2^ = 61%, *P* = 0.04) (Fig. 3C).

To address this heterogeneity, a leave-one-out analysis was performed. Armengou-Garcia 2024 was identified as the primary source of heterogeneity. After excluding this study, heterogeneity was significantly reduced (I^2^ = 49%, *P* = 0.12), and the results remained significant (MD: −95.44 mg, 95% CI: [−143.06, −47.81], *P* < 0.0001) (Supplementary Fig. 6).

### Analysis of AEs at six months

We analyzed only AEs that were reported in at least three studies, while all reported AEs are summarized in Supplementary Table 5.

**Fig. 4:**
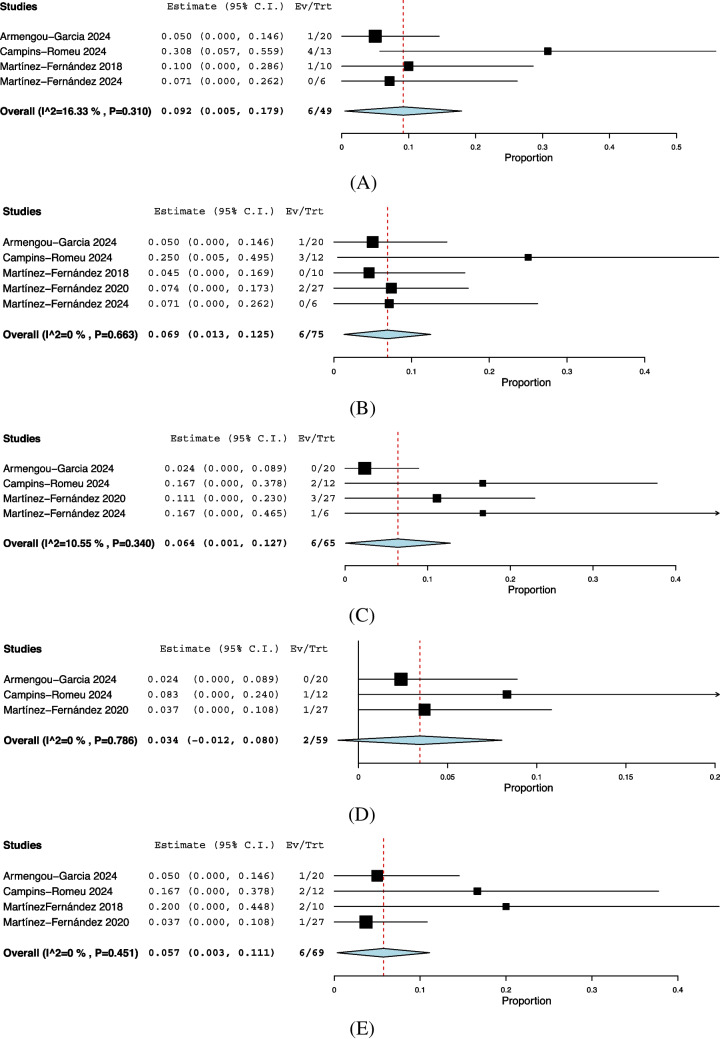
Incidence of common adverse events following FUS-STN. (**A**) Dyskinesia occurred in 9.2% (95% CI: 0.5–17.9%). (**B**) Gait disturbance occurred in 6.9% of patients (95% CI: 1.3–12.5%). (**C**) Dysarthria occurred in 6.4% (95% CI: 0.1–12.7%).(**D**) Facial asymmetry occurred in 3.4% (95% CI: -1.2–8.0%). (**E**) Weight gain occurred in 5.7% (95% CI: 0.3–11.1%)

The incidence of *dyskinesia* was 9.2% (95% CI: [0.5%–17.9%]), occurring in 6 out of 49 patients, with no significant heterogeneity (I^2^ = 16.33%, *P* = 0.310) (Fig. 4A). The incidence of *gait disturbance* was 6.9% (95% CI: [1.3%–12.5%]), occurring in 6 out of 75 patients, with insignificant heterogeneity (I^2^ = 0%, *P* = 0.663) (Fig. 4B). The incidence of *dysarthria* was 6.4% (95% CI: [0.1%–12.7%]), occurring in 6 out of 65 patients, with no significant heterogeneity (I^2^ = 10.55%, *P* = 0.340) (Fig. 4C). The incidence of *facial asymmetry* was 3.4% (95% CI: [-1.2%–8.0%]), occurring in 2 out of 59 patients, with no significant heterogeneity (I^2^ = 0%, *P* = 0.786) (Fig. 4D). Finally, the incidence of *weight gain* was 5.7% (95% CI: [0.3%–11.1%]), occurring in 6 out of 69 patients, with no significant heterogeneity (I^2^ = 0%, *P* = 0.451) (Fig. 4E).

## Discussion

Our meta-analysis demonstrates that MRgFUS-STN may be a safe and effective treatment for selected patients with PD. At six months, pooled results showed a significant improvement in overall motor function, reflected by a mean reduction of 11.05 points in MDS-UPDRS-III scores. This improvement was particularly pronounced in the off-medication state (MD: −13.37 points) compared to the on-medication state (MD: −8.08 points). Importantly, the robustness of these efficacy outcomes was confirmed through sensitivity analyses: excluding the study with a four-month endpoint yielded consistent reductions (off-medication: −12.79; on-medication: −8.25), and omitting the bilateral treatment study further reinforced the magnitude of improvement (off-medication: −15.33; on-medication: −8.84). Collectively, these data reflect a clinically meaningful effect on core motor symptoms like rigidity, bradykinesia, and tremor.

The analysis confirms the focal and unilateral therapeutic effect of the intervention: motor function significantly improved on the treated side (MD: −10.67 points), while the untreated side showed no significant change (MD: 0.00 points). Furthermore, FUS-STN significantly improved secondary outcomes, including activities of daily living (MDS-UPDRS-II, MD: −3.58 points), QoL (PDQ-39, MD: −8.55 points), and reduced dopaminergic medication burden (LEDD, MD: −111.63 mg). These results align with and consolidate findings from prior individual trials that established the efficacy of unilateral FUS-STN [32–35]. This evidence strongly supports the role of FUS-STN as an attractive option for patients who are either ineligible for or unwilling to undergo conventional surgical procedures or device implantation.

Compared to the current gold standard, DBS, FUS-STN offers the unique advantage of being non-incisional, albeit irreversible, thereby avoiding risks associated with invasive intervention and hardware implantation, such as infection, hemorrhage, and mechanical malfunction [36–38]. While DBS offers the benefits of adjustability and reversibility, FUS-STN provides an effective, non-incisional, and irreversible ablative alternative to traditional RF ablation [36, 37].

Based on the focal nature of the intervention, appropriate patient selection is critical. The procedure appears most beneficial for individuals with markedly asymmetrical motor symptoms, relatively early-stage PD, and those who are not suitable candidates for invasive surgery due to comorbidities, advanced age, or personal preference [12, 14, 15, 17].

The overall risk profile for unilateral FUS-STN is favorable, with AEs generally reported as mild and manageable. The most common AEs observed in our pooled analysis were dyskinesia (9.2% incidence), gait disturbance (6.9% incidence), and dysarthria (6.4% incidence). A primary concern with STN ablation is the potential for ablation-induced hemichoreoballismus [16, 18]. This is hypothesized to correlate with the dorsolateral extension of the STN lesion involving the pallidothalamic tract (PTT) [12]. Importantly, post-intervention dyskinesias developed in the on-medication groups were often managed by decreasing the dopaminergic drug dose.

Neurocognitive outcomes also warrant consideration due to the STN's role in executive function and behavior [39, 40]. While most cognitive AEs, such as transient dysarthria or mild verbal fluency decline, have been non-disabling, their occurrence highlights the necessity of comprehensive neuropsychological assessment before and after intervention, particularly if bilateral treatment is considered. Furthermore, research into lesioning strategies, such as minimizing lesion volume, suggests that it may not significantly affect efficacy or the side-effect profile, pointing toward the importance of precise targeting over size alone [13, 15, 16].

Our pooled analysis differs from Cheng et al. [41] in several key methodological respects that materially affect estimates. Notably, Cheng et al. aggregated outcomes over follow-up periods extending to one year, whereas we standardized on a six-month endpoint to minimize time-dependent heterogeneity. Cheng et al. also restricted inclusion to unilateral FUS‐STN cases, while our review incorporated the one available bilateral STN ablation trial. Crucially, we performed fully paired analyses: we computed separate motor‐score changes in both medication-off and on conditions (revealing a significantly greater improvement off medication) and examined scores on the untreated side (confirming essentially no change). Cheng et al. reported MDs for the treated hemibody in off- and on-medication states but did not statistically compare these subgroups or analyze the untreated side. Crucially, our meta‐analysis used empirically derived pre-post CCs (e.g., r≈0.15 for treated-side UPDRS-III and r≈0.716 for the untreated side), in line with Cochrane recommendations [29]. By contrast, Cheng et al. effectively assumed a zero correlation between pre- and post-scores (i.e., treated each score as independent), which Cochrane cautions yields overly conservative variances [29]. Importantly, in their pooled analysis of MDS-UPDRS III subitem scores, Cheng et al. included the same study multiple times in the same subgroup at different follow-up durations, a practice that Cochrane warns against in Chapter 16.5.4 [42]. Re-entering the same participants at multiple timepoints “double-counts” the shared intervention group, inflates sample size, and introduces correlated comparisons without proper statistical adjustment, ultimately biasing effect estimates and precision. Finally, they didn’t meta-analyze AEs and did only descriptive analysis. Collectively, these methodological refinements produce more valid variance estimates, more stable pooled effects, and a more clinically reliable synthesis of MRgFUS-STN outcomes.

In treating PD, MRgFUS-STN has shown significant unilateral motor benefit in selected patients. A recent RCT reported an ~ 8-point greater reduction in MDS-UPDRS-III motor score on the treated side at 4 months with unilateral FUS–STN versus sham [32]. These motor gains translated into improved QoL, albeit at the cost of some reversible AEs, notably speech disturbance, gait ataxia, hemibody weakness, and on-medication dyskinesias, most of which largely resolved by one year [32]. In comparison, bilateral DBS of the STN or GPi yields more global improvement (meta-analyses report ~ 50% UPDRS-III reduction and ~ 20% PDQ-39 quality-of-life gain for STN-DBS) [43], with STN-DBS enabling greater medication reduction and GPi-DBS chosen when cognitive or neuropsychiatric risks are a concern [43, 44]. DBS carries typical surgical risks (hemorrhage ~ 2–3%, infection ~ 5%) and device-related issues, as well as potential neuropsychiatric and speech side effects [43]. Thalamotomy (by RF or focused ultrasound at the Vim nucleus) is highly effective for tremor control: in tremor-dominant PD it powerfully suppresses contralateral tremor and improves daily living activities and QoL [45]. However, it is generally limited to one side due to the high risk of bilateral lesion-induced gait and speech disturbance. Finally, GPi-targeted interventions (unilateral pallidotomy or GPi-DBS) effectively reduce bradykinesia and dyskinesias; posteroventral pallidotomy has level-I evidence of motor benefit comparable to unilateral STN/GPi stimulation [44]. In sum, all modalities improve motor symptoms and QoL but differ in invasiveness and side-effect profiles, and patient factors (symptom laterality, age, cognitive status, and risk tolerance) strongly influence the optimal choice [43, 44].

## Limitations and recommendations

Limitations of this meta-analysis include the small number of available studies (*n* = 5) and the modest overall sample size (*n* = 75). Additionally, most included studies were non-randomized and possessed a moderate risk of bias. While statistical methods addressed heterogeneity, variations across centers in sonication parameters, lesion size, and patient scoring protocols likely remain. The long-term safety and durability of FUS-STN treatment effects beyond six months remain uncertain.

Future research should prioritize:

- Larger, multicenter RCTs with standardized outcome measures and longer follow-up periods (e.g., 2–5 years).
- Further defining the optimal patient population and specific lesion parameters (site, volume).
- Investigating the feasibility and safety of bilateral FUS-STN procedures, especially as symptom progression often leads to contralateral involvement [15]. While staged bilateral procedures have been explored for contralateral symptom progression, the cumulative disruption of frontostriatal networks and risk of persistent speech disturbance need extensive study [8, 15].

## Economic, acceptance, and access considerations

While the clinical evidence for FUS-STN is maturing, its real-world integration faces challenges related to cost, patient acceptance, and equitable access. From an economic standpoint, FUS-STN offers a potentially cost-competitive profile against DBS, primarily by avoiding the substantial long-term expenses associated with hardware, battery replacements, and device-related complications [46, 47]. Patient acceptance is often high, driven by the non-invasive nature of the procedure, which bypasses the psychological barriers and fear of open brain surgery or permanent implanted hardware associated with traditional surgical approaches [48]. However, significant access barriers currently exist. The technology is expensive, leading to limited geographic distribution in specialized centers, meaning patient access is highly dependent on institutional adoption and adequate reimbursement policies. Furthermore, unlike DBS, FUS-STN has technical exclusion criteria, such as an unfavorable skull density ratio, which prevents some otherwise eligible patients from being candidates [49].

## Conclusion

FUS-STN appears to be a safe and effective non-incisional intervention for selected PD patients, offering meaningful improvements in motor symptoms, medication burden, and QoL, particularly in those with asymmetric motor involvement. While mild AEs are relatively common, the overall risk profile remains acceptable, making FUS-STN a promising alternative or adjunct to conventional therapies. The current evidence is limited by small sample size and short to middle follow-up. Further large-scale, long-term studies are warranted to confirm its role in broader clinical practice.

## Supplementary Information

Below is the link to the electronic supplementary material.

Supplementary file1 (PDF 803 KB)

## Data Availability

Data were publicly available.

## Declarations

### Declarations

We confirm that this manuscript complies with all instructions to authors, that all authors meet the authorship criteria and have approved the final version of the manuscript, and the PRISMA checklist was used.

### Generative AI in scientific writing

We declare that we have not used AI in writing any part of this abstract.
