# Supplementary material for: Magnetic resonance-guided focused ultrasound subthalamotomy for Parkinson’s disease: a meta-analysis of effectiveness and safety
Source: Neurosurg Rev. 2026 Feb 7;49(1):201. doi: 10.1007/s10143-025-04045-4 (PMC12882957; doi:10.1007/s10143-025-04045-4)

Magnetic Resonance-Guided Focused Ultrasound Subthalamotomy for Parkinson's Disease: A Meta-Analysis of Effectiveness and Safety

Table of Contents

Supplementary Table 1. Detailed search strategy for each database. 2

Supplementary Table 2. Risk of bias assessment for non-RCTs using the MINORS tool 2

Supplementary Table 3. Risk of bias assessment for RCTs using the RoB 2.0 tool 3

Supplementary Table 4. Risk of bias assessment of the case series studies according to Joanna Briggs Institute (JBI) tool 4

Supplementary Table 5. Summary of adverse events 5

Supplementary Figure 1. Leave-one-out analysis for MDS-UPDRS-III off-medication subgroup 6

Supplementary Figure 2. Comparison of MDS-UPDRS-III reductions between on- and off-medication states 7

Supplementary Figure 3. Sensitivity analysis to the MDS-UPDRS-III after omitting the Martínez-Fernández et al., 2020 study which had four-month endpoint 8

Supplementary Figure 4. Sensitivity analysis to the MDS-UPDRS-III after omitting the Martínez-Fernández et al., 2024 which was bilateral 9

Supplementary Figure 5. Leave-one-out analysis for PDQ-39 10

Supplementary Figure 6. Leave-one-out analysis for LEDD reduction 10

Supplementary Table 1. Detailed search strategy for each database.

Database	Search Terms	Search Field	Search Results
**PubMed**	((“Parkinson's disease” OR Parkinsonism OR PD OR Parkinson*) AND (“Focused ultrasound” OR FUS) AND (Subthalamotomy OR Subthalamic OR “Subthalamic nucleus” OR “Subthalamic lesion” OR STN))	All fields	77
**Scopus**	((“Parkinson's disease” OR Parkinsonism OR PD OR Parkinson*) AND (“Focused ultrasound” OR FUS) AND (Subthalamotomy OR Subthalamic OR “Subthalamic nucleus” OR “Subthalamic lesion” OR STN))	Title/Abstract	66
**WOS**	((“Parkinson's disease” OR Parkinsonism OR PD OR Parkinson*) AND (“Focused ultrasound” OR FUS) AND (Subthalamotomy OR Subthalamic OR “Subthalamic nucleus” OR “Subthalamic lesion” OR STN))	All fields	159
**Cochrane CENTRAL**	((“Parkinson's disease” OR Parkinsonism OR PD OR Parkinson*) AND (“Focused ultrasound” OR FUS) AND (Subthalamotomy OR Subthalamic OR “Subthalamic nucleus” OR “Subthalamic lesion” OR STN))	All fields	13

Supplementary Table 2. Risk of bias assessment for non-RCTs using the MINORS tool

Three non-RCTs were assessed using the MINORS tool and were rated as overall low risk. Armengou-Garcia 2024, Martínez-Fernández 2018, and Campins-Romeu 2024 each scored 1 in the domain of outcome assessment, blinding or measures to minimize bias. Additionally, Campins-Romeu 2024 scored 1 in follow-up duration, Armengou-Garcia 2024 scored 1 in attrition rate reporting or justification, and both Martínez-Fernández 2018 and Campins-Romeu 2024 scored 1 in sample size calculation or power analysis performed before the study began.

**Domain**	Martínez-Fernández 2018	Campins-Romeu 2024	Armengou-Garcia 2024

**Is the study aim or objective stated clearly, specifically, and justified?**	2	2	2
**Were all eligible patients included without selection bias (i.e., consecutively enrolled)?**	2	2	2
**Was data collected according to a pre-established protocol or before outcomes were known?**	2	2	2
**Were outcome measures relevant, clearly defined, and aligned with study objectives?**	2	2	2
**Was outcome assessment blinded or were other measures taken to minimize bias?**	1	1	1
**Was follow-up long enough to assess the main outcomes and adverse events?**	2	1	2
**Was the attrition rate ≤5%, or was it properly described and justified?**	2	2	1
**Was a sample size calculation or power analysis performed before the study began?**	1	1	2
**Total**	14/16	13/16	14/16

Supplementary Table 3. Risk of bias assessment for RCTs using the RoB 2.0 tool

One RCT was assessed using the RoB 2.0 tool and was judged to have a low risk of bias across all domains.

**Study ID**	**D1**	**D2**	**D3**	**D4**	**D5**	**Overall**
Martínez-Fernández 2020	Low	Low	Low	Low	Low	Low

D1 bias arising from the randomization process**;** **D2** bias due to deviations from intended intervention**;** **D3** bias due to missing outcome data**;** **D4** bias in the measurement of the outcome**;** **D5** bias in the selection of the reported result

Supplementary Table 4. Risk of bias assessment of the case series studies according to Joanna Briggs Institute (JBI) tool

One case series was assessed using the JBI tool and was rated as low risk overall; all domains were clearly reported except for consecutive participant inclusion, which was unclear.

	Martínez-Fernández 2024
**1. Were there clear criteria for inclusion in the case series?**	Yes
**2. Was the condition measured in a standard, reliable way for all participants included in the case series?**	Yes
**3. Were valid methods used for the identification of the condition for all participants included in the case series?**	Yes
**4. Did the case series have consecutive inclusion of participants?**	Unclear
**5. Did the case series have a complete inclusion of participants?**	Yes
**6. Was there clear reporting of the demographics of the participants in the study?**	Yes
**7. Was there clear reporting of clinical information of the participants?**	Yes
**8. Were the outcomes or follow-up results of cases clearly reported?**	Yes
**9. Was there clear reporting of the presenting site (s)/clinic (s) demographic information?**	Yes
**10. Was statistical analysis appropriate?**	Yes
**Overall**	**9 out of 10**

Supplementary Table 5. Summary of adverse events

AE	Incidence in FUS-STN (Events/Total, % of incidence)
Mild AEs	13/32, 40.63%
Moderate AEs	0/32, 0%
Severe AEs	0/32, 0%
Dyskinesia	6/49, 12.24%
Gait disturbance	6/75, 8.00%
Dysarthria	6/65, 9.23%
Paresthesia	1/32, 3.13%
Weakness	2/32, 6.25%
Facial asymmetry	2/59, 3.39%
Weight gain	6/69, 8.70%
Nausea	7/37, 18.92%
Dizziness	13/27, 48.15%
Behavioral changes	0/30, 0%
Headache	5/27, 18.52%

Supplementary Figure 1. Leave-one-out analysis for MDS-UPDRS-III off-medication subgroup

Exclusion of *Martínez-Fernández 2024* markedly reduced heterogeneity ($I^2 = 31\%$, $P = 0.23$) while maintaining significant improvement (MD: –15.33; 95% CI: –18.16 to –12.51; $P < 0.00001$).

Study or Subgroup	Mean Difference	SE	Weight	Mean Difference IV, Random, 95% CI
**1.8.1 MDS-UPDRS-III (On-Medication)**				
Campins-Romeu 2024	-16.84	4.70059044	8.6%	-16.84 [-26.05, -7.63]
Martínez-Fernández 2018	-7	2.19879558	14.5%	-7.00 [-11.31, -2.69]
Martínez-Fernández 2020	-8.4	0.90066642	17.3%	-8.40 [-10.17, -6.63]
Martínez-Fernández 2024	-3	3.73431759	10.6%	-3.00 [-10.32, 4.32]
**Subtotal (95% CI)**			**51.0%**	**-8.08 [-11.30, -4.86]**
Heterogeneity: Tau² = 4.86; Chi² = 5.66, df = 3 (P = 0.13); I² = 47%				
Test for overall effect: Z = 4.91 (P < 0.00001)				
**1.8.2 MDS-UPDRS-III (Off-Medication)**				
Armengou-Garcia 2024	-19.9	3.24902042	11.8%	-19.90 [-26.27, -13.53]
Campins-Romeu 2024	-18.5	5.96029157	6.5%	-18.50 [-30.18, -6.82]
Martínez-Fernández 2018	-11.5	2.65761246	13.3%	-11.50 [-16.71, -6.29]
Martínez-Fernández 2020	-15.2	0.8506294	17.4%	-15.20 [-16.87, -13.53]
**Subtotal (95% CI)**			**49.0%**	**-15.33 [-18.16, -12.51]**
Heterogeneity: Tau² = 2.86; Chi² = 4.34, df = 3 (P = 0.23); I² = 31%				
Test for overall effect: Z = 10.63 (P < 0.00001)				
**Total (95% CI)**			**100.0%**	**-11.95 [-15.68, -8.23]**
Heterogeneity: Tau² = 20.02; Chi² = 49.19, df = 7 (P < 0.00001); I² = 86%				
Test for overall effect: Z = 6.29 (P < 0.00001)				
Test for subgroup differences: Chi² = 10.99, df = 1 (P = 0.0009), I² = 90.9%				

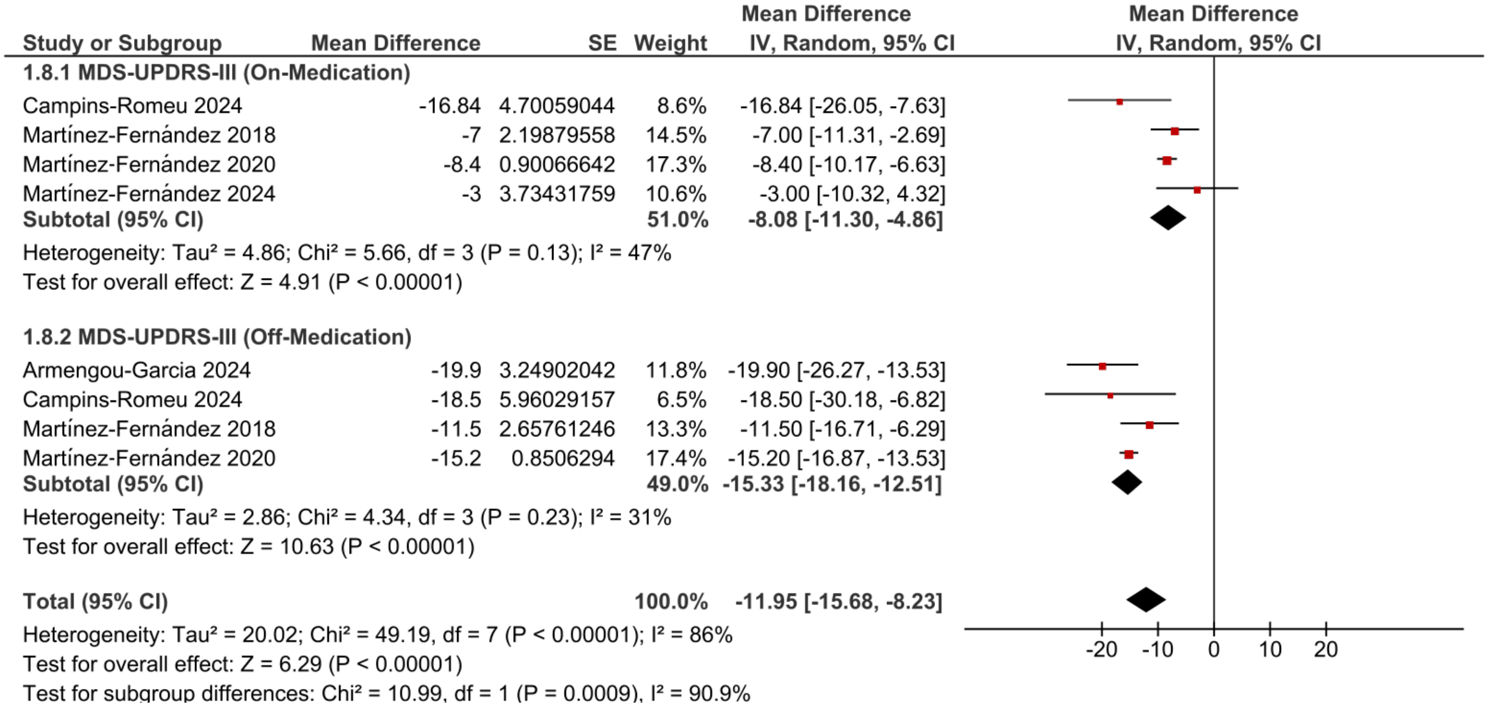

Supplementary Figure 2. Comparison of MDS-UPDRS-III reductions between on- and off-medication states

The off-medication subgroup showed a significantly greater motor score reduction than the on-medication subgroup (P = 0.0009), confirming the procedure’s efficacy independent of dopaminergic therapy.

Study or Subgroup	Mean Difference	SE	Weight	Mean Difference IV, Random, 95% CI
**1.8.1 Treated Side [MDS-UPDRS-III (On-Medication)]**				
Campins-Romeu 2024	-6.5	2.17286293	15.7%	-6.50 [-10.76, -2.24]
Martínez-Fernández 2018	-6.1	1.36400147	18.6%	-6.10 [-8.77, -3.43]
Martínez-Fernández 2024	-11.97	2.50984727	14.4%	-11.97 [-16.89, -7.05]
**Subtotal (95% CI)**			**48.8%**	**-7.74 [-11.04, -4.45]**
Heterogeneity: Tau² = 4.61; Chi² = 4.37, df = 2 (P = 0.11); I² = 54%				
Test for overall effect: Z = 4.60 (P < 0.00001)				
**1.8.2 Treated Side [MDS-UPDRS-III (Off-Medication)]**				
Armengou-Garcia 2024	-13.7	1.35724179	18.6%	-13.70 [-16.36, -11.04]
Campins-Romeu 2024	-12.83	1.97440603	16.4%	-12.83 [-16.70, -8.96]
Martínez-Fernández 2024	-15	2.05187394	16.1%	-15.00 [-19.02, -10.98]
**Subtotal (95% CI)**			**51.2%**	**-13.78 [-15.71, -11.86]**
Heterogeneity: Tau² = 0.00; Chi² = 0.59, df = 2 (P = 0.75); I² = 0%				
Test for overall effect: Z = 14.03 (P < 0.00001)				
**Total (95% CI)**			**100.0%**	**-10.97 [-14.28, -7.66]**
Heterogeneity: Tau² = 13.46; Chi² = 25.81, df = 5 (P < 0.0001); I² = 81%				
Test for overall effect: Z = 6.50 (P < 0.00001)				
Test for subgroup differences: Chi² = 9.61, df = 1 (P = 0.002), I² = 89.6%				

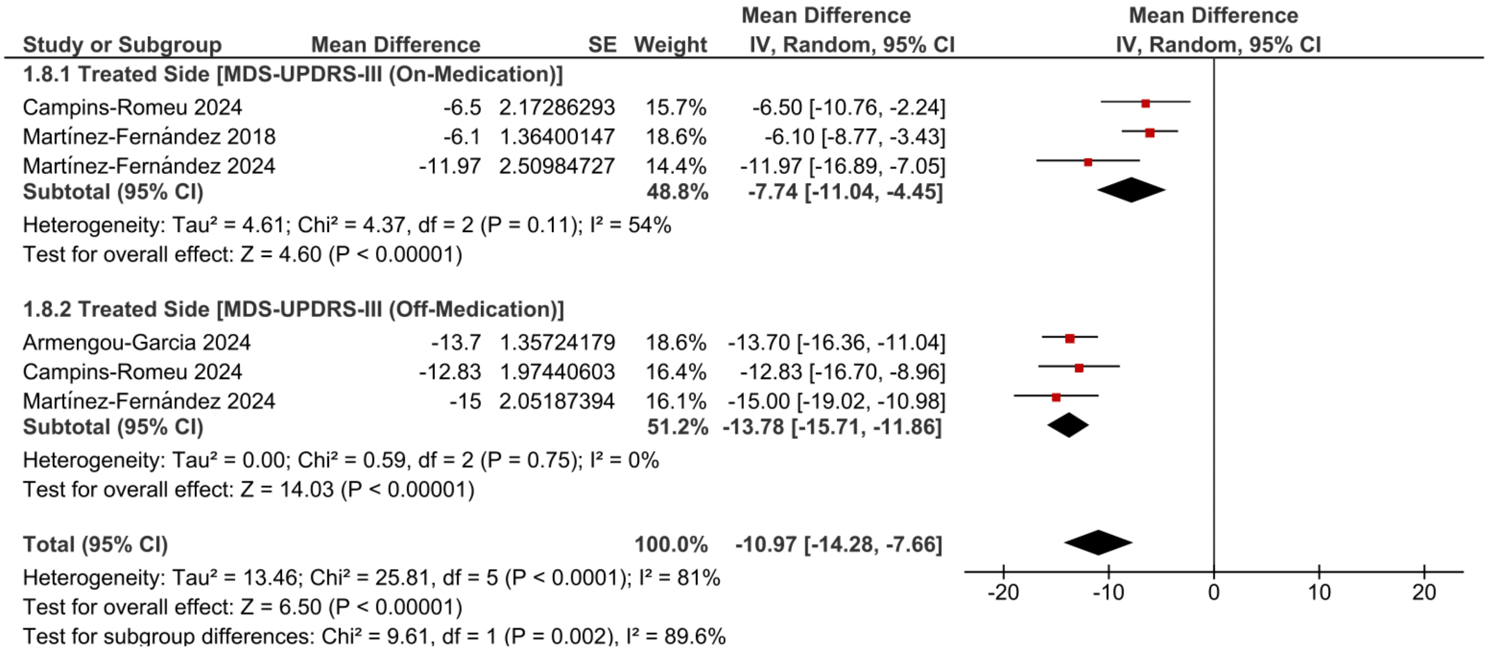

Supplementary Figure 3. Sensitivity analysis to the MDS-UPDRS-III after omitting the Martínez-Fernández et al., 2020 study which had four-month endpoint

Study or Subgroup	MD	SE	Weight	Mean difference IV, Random, 95% CI
**1.3.1 MDS-UPDRS-III (On-Medication)**				
Campins-Romeu 2024	-16.84	4.70059044	12.0%	-16.84 [-26.05 , -7.63]
Martinez-Fernandez 2018	-7	2.19879558	17.7%	-7.00 [-11.31 , -2.69]
Martinez-Fernandez 2024	-3	3.73431759	14.2%	-3.00 [-10.32 , 4.32]
**Subtotal (Wald[a])**			**43.9%**	**-8.25 [-14.70 , -1.80]**
Test for overall effect: Z = 2.51 (P = 0.01)				
Heterogeneity: Tau^2 (DL[b]) = 20.37; Chi^2 = 5.45, df = 2 (P = 0.07); I^2 = 63%				
**1.3.2 MDS-UPDRS-III (Off-Medication)**				
Armengou-Garcia 2024	-19.9	3.24902042	15.3%	-19.90 [-26.27 , -13.53]
Campins-Romeu 2024	-18.5	5.96029157	9.6%	-18.50 [-30.18 , -6.82]
Martinez-Fernandez 2018	-11.5	2.65761246	16.7%	-11.50 [-16.71 , -6.29]
Martinez-Fernandez 2024	-2.73	3.57264872	14.5%	-2.73 [-9.73 , 4.27]
**Subtotal (Wald[a])**			**56.1%**	**-12.79 [-20.40 , -5.19]**
Test for overall effect: Z = 3.30 (P = 0.0010)				
Heterogeneity: Tau^2 (DL[b]) = 45.46; Chi^2 = 13.82, df = 3 (P = 0.003); I^2 = 78%				
**Total (Wald[a])**			**100.0%**	**-10.83 [-15.84 , -5.82]**

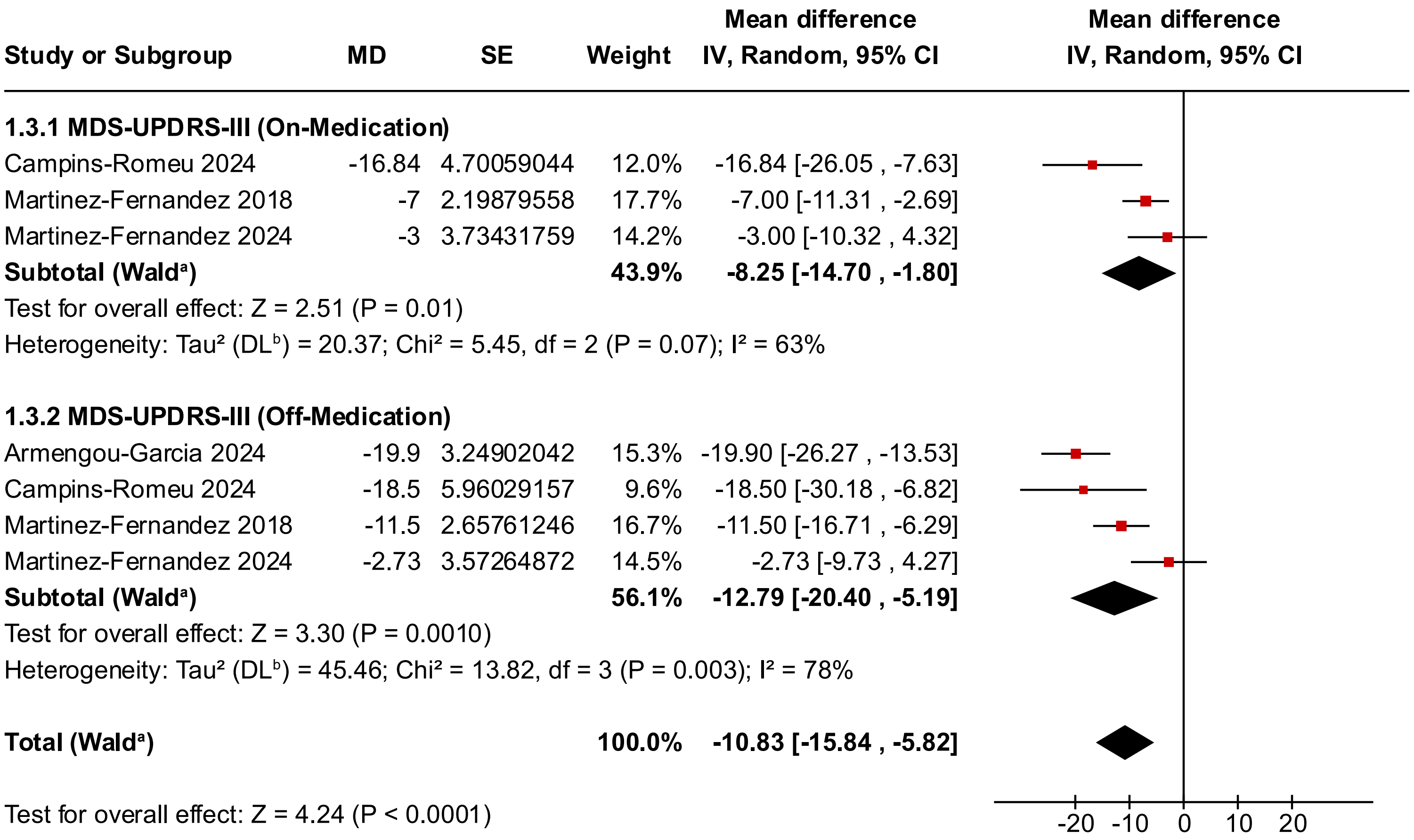

Test for overall effect: Z = 4.24 (P < 0.0001)
Test for subgroup differences: Chi^2 = 0.80, df = 1 (P = 0.37), I^2 = 0%
Heterogeneity: Tau^2 (DL[b]) = 32.12; Chi^2 = 23.27, df = 6 (P = 0.0007); I^2 = 74%

Footnotes

[a]CI calculated by Wald-type method.

[b]Tau^2 calculated by DerSimonian and Laird method.

Supplementary Figure 4. Sensitivity analysis to the MDS-UPDRS-III after omitting the Martínez-Fernández et al., 2024 which was bilateral

Study or Subgroup	MD	SE	Weight	Mean difference IV, Random, 95% CI
**1.2.1 MDS-UPDRS-III (On-Medication)**				
Campins-Romeu 2024	-16.84	4.70059044	9.4%	-16.84 [-26.05 , -7.63]
Martinez-Fernandez 2018	-7	2.19879558	16.3%	-7.00 [-11.31 , -2.69]
Martinez-Fernandez 2020	-8.4	0.90066642	19.6%	-8.40 [-10.17 , -6.63]
**Subtotal (Wald[a])**			**45.3%**	**-8.84 [-12.05 , -5.63]**
Test for overall effect: Z = 5.40 (P < 0.00001)				
Heterogeneity: Tau² (DL[b]) = 3.77; Chi² = 3.62, df = 2 (P = 0.16); I² = 45%				
**1.2.2 MDS-UPDRS-III (Off-Medication)**				
Armengou-Garcia 2024	-19.9	3.24902042	13.1%	-19.90 [-26.27 , -13.53]
Campins-Romeu 2024	-18.5	5.96029157	7.1%	-18.50 [-30.18 , -6.82]
Martinez-Fernandez 2018	-11.5	2.65761246	14.9%	-11.50 [-16.71 , -6.29]
Martinez-Fernandez 2020	-15.2	0.8506294	19.7%	-15.20 [-16.87 , -13.53]
**Subtotal (Wald[a])**			**54.7%**	**-15.33 [-18.16 , -12.51]**
Test for overall effect: Z = 10.63 (P < 0.00001)				
Heterogeneity: Tau² (DL[b]) = 2.86; Chi² = 4.34, df = 3 (P = 0.23); I² = 31%				
**Total (Wald[a])**			**100.0%**	**-12.98 [-16.81 , -9.15]**

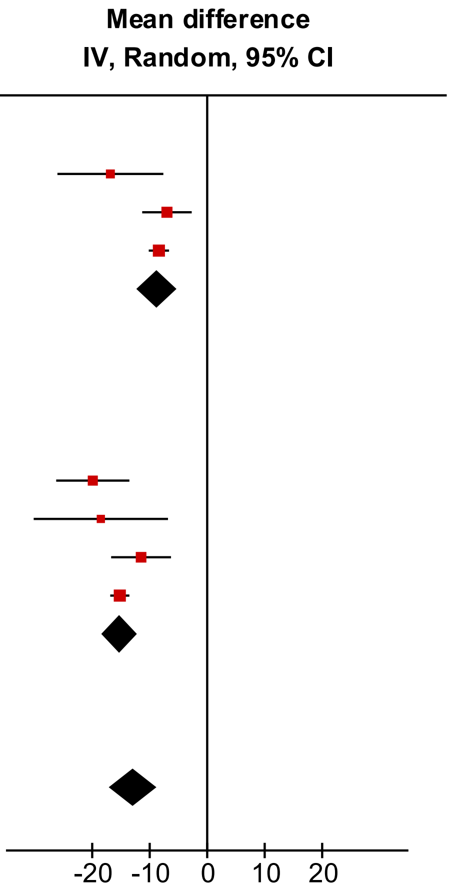

Test for overall effect: Z = 6.64 (P < 0.00001)

Test for subgroup differences: Chi² = 8.86, df = 1 (P = 0.003), I² = 88.7%

Heterogeneity: Tau² (DL[b]) = 18.63; Chi² = 43.50, df = 6 (P < 0.00001); I² = 86%

Footnotes

[a]CI calculated by Wald-type method.

[b]Tau² calculated by DerSimonian and Laird method.

Supplementary Figure 5. Leave-one-out analysis for PDQ-39

Removal of *Martínez-Fernández 2018* reduced heterogeneity ($I^2 = 56\%$, $P = 0.11$), with consistent improvement in PDQ-39 (MD: –10.72; 95% CI: –16.07 to –5.36; $P < 0.0001$), indicating robust improvement in quality of life.

Study or Subgroup	Mean Difference	SE	Weight	Mean Difference IV, Random, 95% CI
**1.8.1 The Parkinson's Disease Questionnaire (PDQ-39)**				
Campins-Romeu 2024	-16.77	4.48716629	22.8%	-16.77 [-25.56, -7.98]
Martínez-Fernández 2020	-7.4	1.58193974	49.3%	-7.40 [-10.50, -4.30]
Martínez-Fernández 2024	-11.64	3.76364099	27.9%	-11.64 [-19.02, -4.26]
**Subtotal (95% CI)**			**100.0%**	**-10.72 [-16.07, -5.36]**
Heterogeneity: Tau² = 12.63; Chi² = 4.50, df = 2 (P = 0.11); I² = 56%				
Test for overall effect: Z = 3.92 (P < 0.0001)				

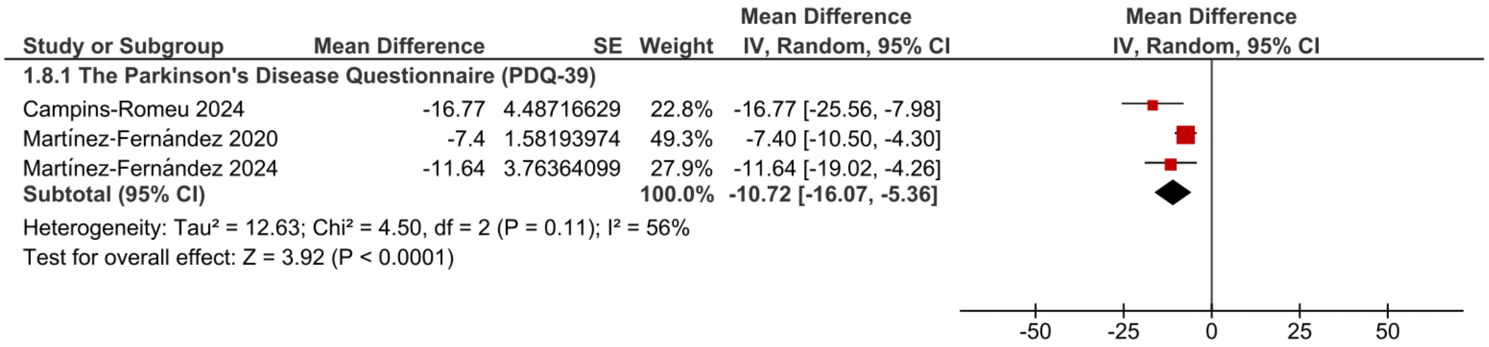

Supplementary Figure 6. Leave-one-out analysis for LEDD reduction

Exclusion of *Armengou-Garcia 2024* reduced heterogeneity ($I^2 = 49\%$, $P = 0.12$) without affecting significance (MD: –95.44 mg; 95% CI: –143.06 to –47.81; $P < 0.0001$), confirming the consistency of reduced medication requirements after FUS-STN.

Study or Subgroup	Mean Difference	SE	Weight	Mean Difference IV, Random, 95% CI
**2.8.1 Levodopaequivalentdailydose(LEDD),mg**				
Campins-Romeu 2024	-89.33	23.65028775	35.3%	-89.33 [-135.68, -42.98]
Martínez-Fernández 2018	-168.3	41.78613741	20.7%	-168.30 [-250.20, -86.40]
Martínez-Fernández 2020	-94.5	27.89948951	31.2%	-94.50 [-149.18, -39.82]
Martínez-Fernández 2024	3.26	59.25968885	12.8%	3.26 [-112.89, 119.41]
**Subtotal (95% CI)**			**100.0%**	**-95.44 [-143.06, -47.81]**
Heterogeneity: Tau² = 1111.47; Chi² = 5.88, df = 3 (P = 0.12); I² = 49%				
Test for overall effect: Z = 3.93 (P < 0.0001)				

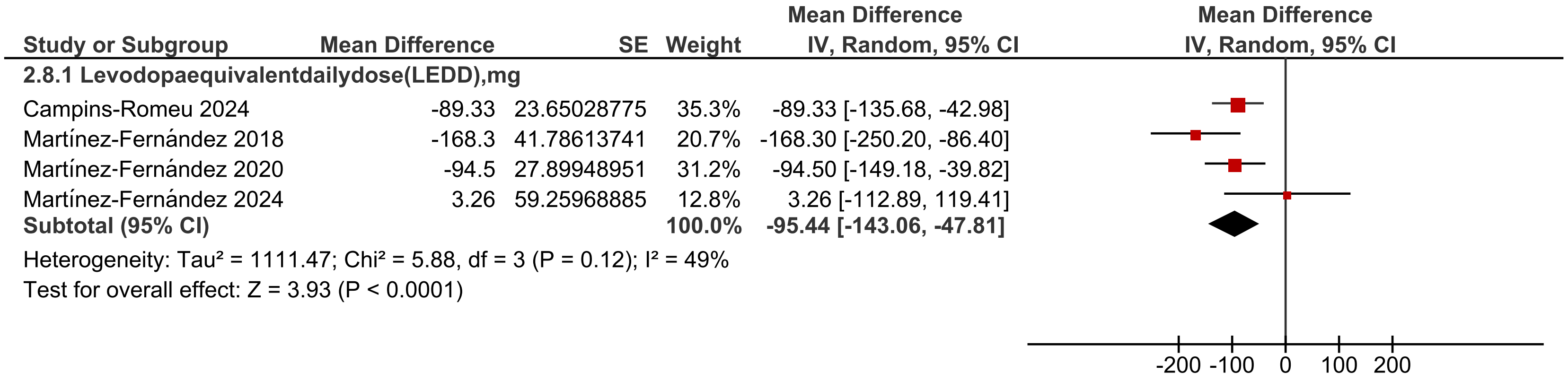

Supplement: Supplementary file 1 — Supplementary file1 (PDF 803 KB) [file 10143_2025_4045_MOESM1_ESM.pdf]
